# Skeleton Enhanced Dispersed Lubricant Particle Based Triboelectric Nanogenerator for Droplet Energy Harvesting

**DOI:** 10.1002/advs.202505363

**Published:** 2025-05-28

**Authors:** Changjun Yang, Yan Wang, Yamei Wang, Zelinlan Wang, Yurun Guo, Liwen Zhang, Xiaolin Liu, Huawei Chen

**Affiliations:** ^1^ School of Mechanical Engineering and Automation Beihang University Beijing 100191 China; ^2^ School of Mechanical Engineering Dalian University of Technology Dalian 116024 China; ^3^ Beijing Advanced Innovation Center for Biomedical Engineering Beihang University Beijing 100191 China

**Keywords:** droplet energy harvesting, high transfer charge density, skeleton‐enhanced lubricant particle, slippery surface, triboelectric nanogenerator

## Abstract

Liquid–solid triboelectric nanogenerators (LS‐TENGs) can be widely utilized for droplet energy harvesting, in which slippery modification of triboelectric layer is crucial for output enhancement. However, classical slippery lubricant‐infused surfaces suffer from the blocked triboelectric effect and the poor durability. Herein, a controllable phase separation method is reported to disperse skeleton‐enhanced lubricant particles on triboelectric layer, leading to the development of a stretchable slippery triboelectric nanogenerator (SS‐TENG) based on a modified slippery triboelectric layer and a liquid metal electrode. The dispersed lubricant particles (DLPs) ensure triboelectric effect between droplet and triboelectric layer, in addition to improving energy harvesting and charge transfer efficiencies. As a result, the open circuit voltage significantly increases from 0.9 to 14.4 V, with a transfer charge density of 6.95 × 10^−3^ C m^−2^ L^−1^. The embedded skeleton within lubricant particle significantly improves the durability of triboelectric layer, ensuring nearly no decline in output performance of SS‐TENG during long‐term operation. Furthermore, the SS‐TENG exhibits stable output even under 300% stretching, as the DLPs remain firmly anchored to triboelectric layer during deformation. Owing to its excellent triboelectric performance, durability, and flexibility, the SS‐TENG can be integrated into various objects to harvest raindrop energy and power electronic devices.

## Graphical abstract

A stretchable, slippery triboelectric nanogenerator (SS‐TENG) is presented for efficient droplet energy harvesting. A novel phase separation method is created to prepare a durable, flexible triboelectric layer with skeleton‐embedded, dispersed lubricant particles. The SS‐TENG exhibits high triboelectricity, durability, and flexibility, demonstrating great potential for harvesting droplet energy and powering IoT devices in diverse applications.

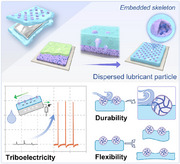

## 1 Introduction

The Internet of Things (IoT) holds significant potential for applications in urban life, agricultural production, factory supervision, disaster monitoring, and border security due to its intelligent, informative, and networked capabilities.^[^ ^1^, ^2^ ^]^ However, its widespread and remote deployment is hindered by challenges such as high costs, limited coverage, and the need for regular recharging or battery replacement in traditional wired or battery‐powered solutions.^[^ ^3^, ^4^, ^5^ ^]^ While the adoption of clean energy sources, such as solar energy,^[^ ^6^, ^7^ ^]^ wind energy,^[^ ^8^, ^9^ ^]^ and mechanical energy,^[^ ^10^, ^11^ ^]^ has partially addressed energy supply issues. But these energy harvesting systems often suffer from drawbacks such as large size, complex structures, and high costs. Water energy as a renewable energy source is abundantly available in nature in the form of stream, droplet, etc.^[^ ^12^, ^13^, ^14^ ^]^ However, conventional hydroelectric power generation systems have primarily focused on capturing the stream energy, largely overlooking the potential of droplet energy.^[^ ^15^, ^16^, ^17^ ^]^

Research on Liquid–solid triboelectric nanogenerators (LS‐TENGs) has demonstrated their potential for harvesting droplet energy and powering electronic devices.^[^ ^18^, ^19^, ^20^, ^21^, ^22^ ^]^ However, the output performance of LS‐TENGs needs further improvement to meet the energy requirements of modern electronics.^[^ ^23^, ^24^, ^25^ ^]^ Lubricating treatment of triboelectric layer improves droplet spreading and separation efficiency, which is crucial for enhancing the charge‐shield release efficiency between the droplet and the triboelectric layer, thereby improving the LS‐TENG generation,^[^ ^26^, ^27^ ^]^ and it can be achieved by infusing lubricant into the porous triboelectric layer surface.^[^ ^28^, ^29^, ^30^, ^31^ ^]^ Nevertheless, the triboelectric effect between droplet and triboelectric layer was blocked by the liquid lubricant, negatively impacting output performance.^[^ ^29^, ^30^ ^]^ Additionally, the durability of the triboelectric layer is limited, as the liquid lubricant tends to deplete after multiple droplet cycles.^[^ ^28^, ^29^, ^30^ ^]^ Furthermore, current research has not adequately addressed the flexibility of LS‐TENGs, which is essential for integrating them with diverse objects.^[^ ^28^, ^32^ ^]^

In this research, we present a stretchable slippery triboelectric nanogenerator (SS‐TENG) with exceptional electrical and mechanical properties. A controllable phase separation method was developed to fabricate a dispersed lubricant particle (DLP) modified slippery triboelectric layer, where lubricant particles are dispersed on the triboelectric layer to create a slippery interface. The durability of the layer was enhanced by a skeleton structure embedded within the lubricant particles. The DLPs not only preserve the triboelectric effect between droplets and the triboelectric layer but also simultaneously improve droplet mechanical energy harvesting capture efficiency and charge transfer efficiency. As a result, the SS‐TENG exhibits significantly higher output performance compared to unmodified LS‐TENG. The SS‐TENG demonstrates remarkable durability due to the robust skeleton‐enhanced lubricant particles that protect the triboelectric layer from flush delamination. Additionally, it maintains excellent flexibility due to the DLPs on the triboelectric layer and the shape‐adaptable liquid metal (LM) electrode. In an application experiment, the SS‐TENG successfully powered a calculator, demonstrating its potential for integration into various objects to harvest droplet energy and supply power for IoT devices.

## 2 Results and Discussion

### 2.1 Advantages of DLP on Triboelectric Layer for SS‐TENG

**Figure 1:**
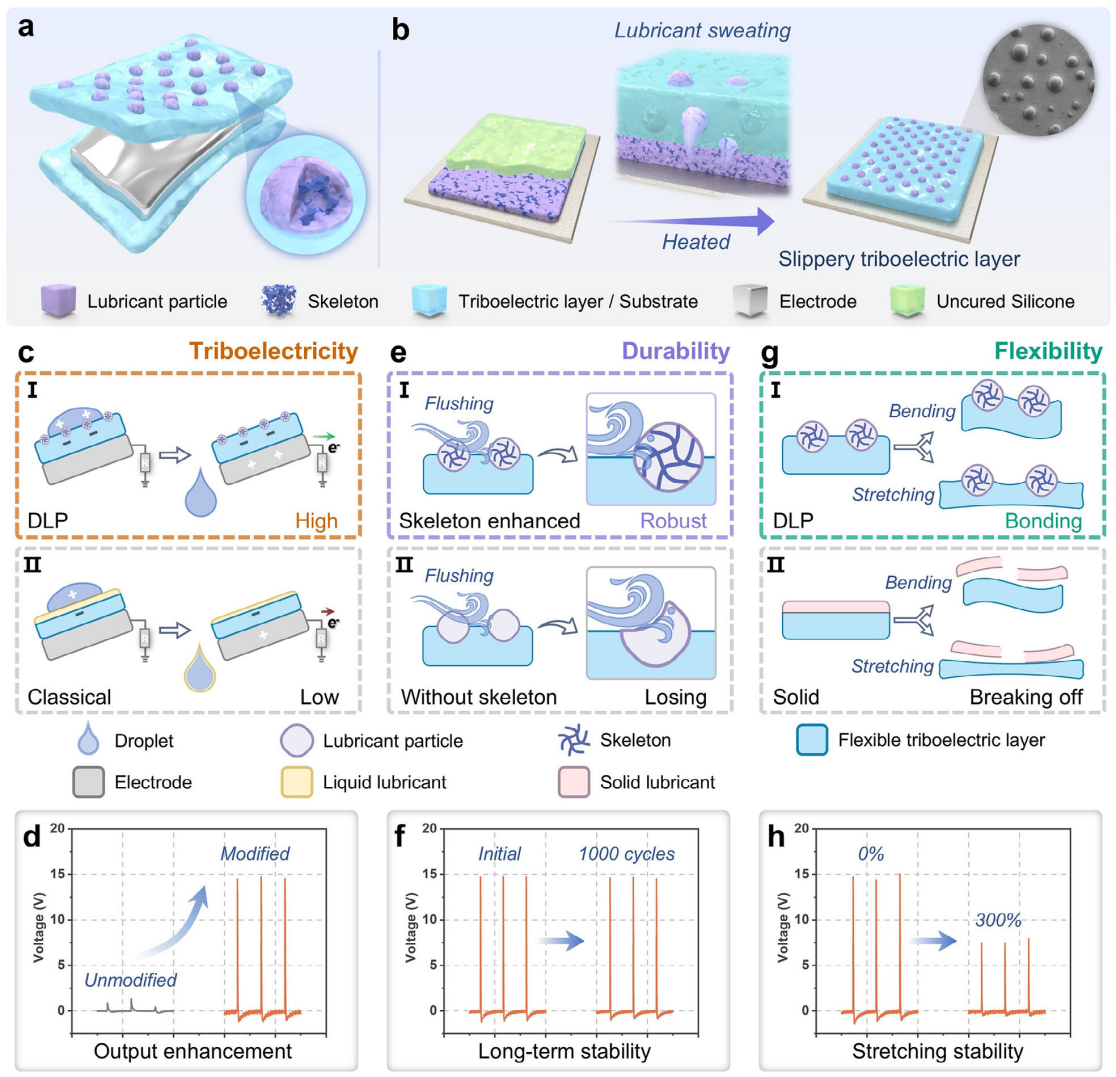
Advantages of SS‐TENG based on dispersed lubricant particles (DLPs) on triboelectric layer. a) Schematic diagram of SS‐TENG, consisting of a top triboelectric layer, a middle electrode layer, and a bottom substrate layer, in which the lubricant particles with embedded enhanced‐skeleton disperse on the triboelectric layer. b) Preparation of the slippery triboelectric layer using a controllable phase separation method. c) DLPs enhance both droplet transfer efficiency and the triboelectric effect between the droplet and the triboelectric layer (I); But in conventional researches, the liquid lubricant blocks the triboelectric effect and depleted over time during operation (II). d) Significant improvement in output performance achieved by incorporating DLPs on the triboelectric layer. e) Enhanced flushing durability of the lubricant triboelectric layer due to the skeleton within the DLP (I), compared to a lubricant triboelectric layer without a skeleton (II). f) The SS‐TENG demonstrates excellent long‐term stability. g) Flexibility of the triboelectric layer is maintained as the lubricant particles remain bonded to the surface even under bending or stretching (I); In contrast, solid lubricant surface tends to crack and detach during deformation (II). h) Stable output performance of the SS‐TENG even under a stretched state.

The SS‐TENG was composed by the top triboelectric layer, middle electrode layer, and bottom substrate layer (**Figure** **1**a), in which the lubricant particles were dispersed on the triboelectric layer with the robust skeleton formed inside the lubricant particle (inset diagram). The preparation process of SS‐TENG is illustrated in Figure S1 (Supporting Information). The slippery triboelectric layer was prepared by a controllable phase separation method (Figure 1b), successfully forming lubricant particles on its surface (inset diagram). The DLPs enhanced the contact‐separation efficiency between the droplet and the triboelectric layer, preventing droplet pinning on triboelectric layer after separation (Figure 1c,I). Additionally, the DLPs preserved the triboelectric effect between the droplet and the triboelectric layer while improving droplet transfer efficiency. In conventional researches, the slippery triboelectric layer was fabricated by infusing lubricant into a porous surface (Figure 1c,II). However, the liquid lubricant blocked the triboelectric effect, negatively impacting output performance. Moreover, the liquid lubricant was prone to depletion after multiple droplet‐flushing cycles, leading to the failure of the slippery properties. In contrast, the lubricant particles dispersed on triboelectric layer significantly improved the peak voltage of the SS‐TENG, achieving a performance ≈15 times higher than that of an LS‐TENG with an unmodified triboelectric layer (Figure 1d). The skeleton within the lubricant particles significantly enhanced the flush durability of the slippery triboelectric layer, as the particles remained intact on the surface even after 24 h of continuous droplet flushing (Figure 1e,I). In contrast, lubricant particles without a skeleton were completely depleted after just 1 h of droplet flushing (Figure 1e,II). As a result, the SS‐TENG incorporating skeleton‐enhanced lubricant particles exhibited nearly no decline in output performance even after 1000 working cycles (Figure 1f). The DLPs remained firmly bonded to the triboelectric layer even under bending or stretching, ensuring the layer's flexibility (Figure 1g,I). For comparison, a solid slippery triboelectric layer was prepared using the same method but with paraffin wax replacing the lubricant component, forming a solid lubricant layer on the triboelectric surface. However, under bending or stretching, the solid lubricant layer tended to crack and peel off, resulting in a loss of slipperiness (Figure 1g,II). In contrast, the SS‐TENG with skeleton‐enhanced lubricant particles maintained stable output performance even at a stretching rate of 300% (Figure 1h). In summary, the SS‐TENG simultaneously demonstrated excellent triboelectricity, durability, and flexibility.

### 2.2 Characteristics of SS‐TENG and Triboelectric Layer

**Figure 2:**
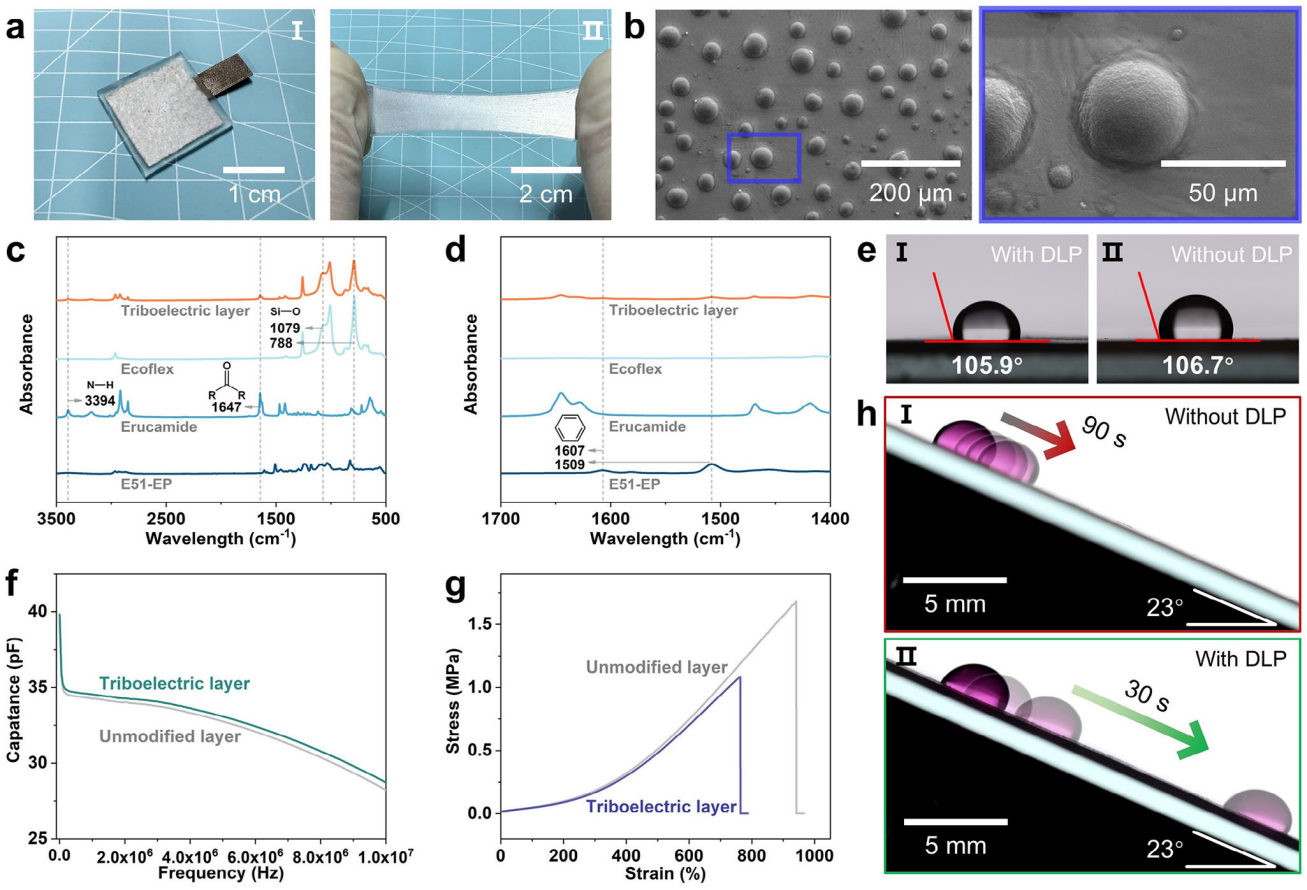
Illustration and characterization of SS‐TENG. a) Digital photograph of SS‐TENG in initial state (I) and stretched state (II). b) SEM images of triboelectric layer, showing DLPs bonded on the surface. c,d) FTIR spectrum comparison between triboelectric layer, Ecoflex polymer, Erucamide, and E51‐EP. e) Water contact angles of DLP‐modified triboelectric layer (I) and pristine Ecoflex polymer (II). f) Capacitance comparison between triboelectric layer and unmodified layer. g) Stress–strain curve comparison between triboelectric layer and unmodified layer. h) Optical images of droplet slippage on pristine Ecoflex polymer (I) and DLP‐modified triboelectric layer (II).

A SS‐TENG sample with a power generation area of 1.5 × 1.5 cm^2^ was prepared (**Figure** **2**a,I), demonstrating excellent stretchability (Figure 2a,II). Scanning electron microscope (SEM) images reveal that (DLPs) are firmly bonded to the Ecoflex polymer surface (Figure 2b). The EDS images of elemental C (Figure S2a, Supporting Information) and Si (Figure S2b, Supporting Information) further confirm the successful formation of DLPs on triboelectric layer. The embedded skeleton structure in DLPs was characterized by the hydrothermal flushing tests. (Figure S3, Supporting Information.) The mechanism of the controllable phase separation method was further elucidated through a series of systematic experiments. (Figure S4, Supporting Information) Fourier transform infrared (FTIR) spectroscopy was used to analyze the composition of the triboelectric layer. Peaks at 788 and 1079 cm^−1^ correspond to the Si─O stretching vibrations of Ecoflex; Peaks at 1647 and 3394 cm^−1^ are attributed to the carbonyl unit (amide groups) and N─H (amino unit) of erucamide, respectively (Figure 2c). Additionally, peaks at 1509 and 1607 cm^−1^ are assigned to the C─H vibrations of the benzene ring in the E51‐EP skeleton (Figure 2d), with detailed views provided in Figure S5a,b (Supporting Information). These FTIR results confirm the successful preparation of DLPs on the layer.

Water contact angle measurements show that the hydrophobicity of the DLP‐modified triboelectric layer (105.9°, Figure 2e,I) is nearly identical to that of the pristine Ecoflex polymer (without DLP‐modified) (106.7°, Figure 2e,II), indicating that the DLPs do not significantly alter the surface hydrophobicity. This slight hydrophobicity effectively reduces droplet pinning, ensuring stable output performance. Capacitance (*C*) measurements of the triboelectric layer (300 µm thick) and unmodified layer were conducted over a frequency range of 1 kHz to 10 MHz (Figure 2f). The capacitance of the triboelectric layer remains nearly unchanged compared to the unmodified layer, indicating that the DLPs do not adversely affect the output performance, as further detailed in Supporting Information. The Young's modulus of the triboelectric layer is 0.032 MPa, similar to that of the unmodified layer (Figure 2g), while the breaking elongation reaches 763%, demonstrating excellent stretchability and flexibility. Droplet (18 µL) behavior analysis shows that droplets slide slowly on the pristine Ecoflex polymer (without DLP‐modified) (Figure 2h,I) but rapidly on the DLP‐modified triboelectric layer (Figure 2h,II) under the same conditions. This enhanced sliding behavior is critical for improving the contact‐separation efficiency between droplets and the triboelectric layer.

### 2.3 Performance Evaluation of SS‐TENG

#### 2.3.1 Performance of SS‐TENG Prepared via Control Phase Separation

Before conducting system tests on the SS‐TENG, it is essential to analyze the factors influencing its output performance. The operating principle of the SS‐TENG in this research is based on Maxwell's displacement current,^[^ ^33^, ^34^ ^]^ and the short circuit current (*I*_SC_) and open circuit voltage (*V*_OC_) are expressed as:

(1) $$I_{\mathrm{SC}}=\frac{dQ}{dt}=A\frac{d\sigma_{1}\left(z,t\right)}{dt}$$

(2) $$V_{\mathrm{OC}}=RA\frac{d\sigma_{1}\left(z,t\right)}{dt}+A\frac{\sigma_{1}\left(z,t\right)}{C}$$

where *Q* is the transfer charge in electrode, *t* is the contact‐separation time of the droplet on triboelectric layer, *A* is the spreading area of the droplet on triboelectric layer, *σ_I_* is the free charge density in electrode, *R* is the resistance of external load, *C* is the capacitance of triboelectric layer. The detailed derivation process is provided in the Supporting Information. The working principle of SS‐TENG is illustrated in Figure S6 (Supporting Information).

**Figure 3:**
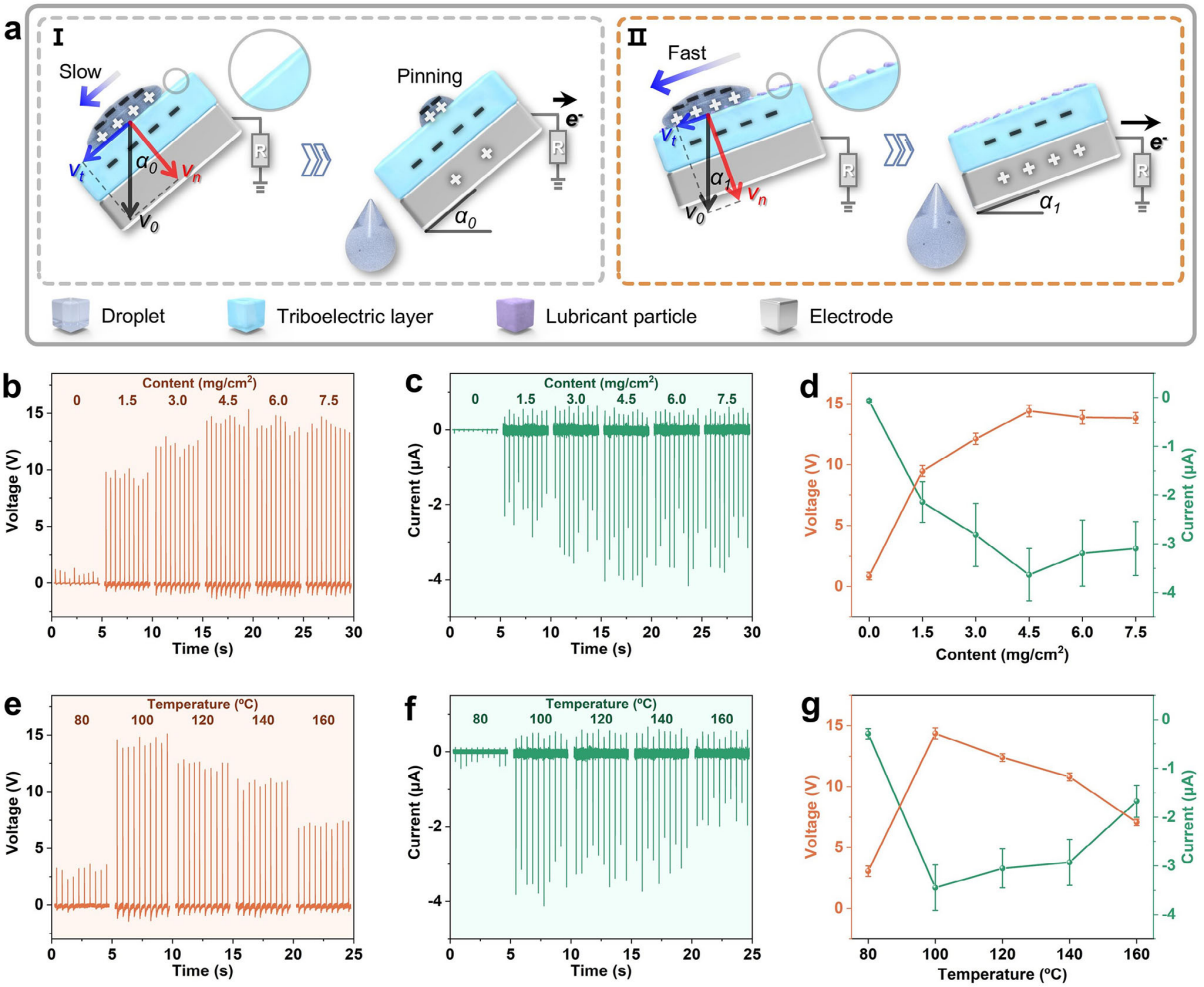
Output of SS‐TENG based on the controllable phase separation preparation. a) Schematic diagram of contact‐separation and charge transfer of the droplet with the unmodified triboelectric layer (I) and the DLP‐modified triboelectric layer (II). b) *V*_OC_ and c) *I*_SC_ under different DLP precursor contents (0–7.5 mg cm^−2^) at a heating temperature of 100 °C, d) reaching peak values at a content of 4.5 mg cm^−2^. e) *V*_OC_ and f) *I*_SC_ at different heating temperatures (80–160 °C) with a DLP precursor content of 4.5 mg cm^−2^, g) reaching peak values at a temperature of 100 °C. Error bars represent the standard deviation of measurements.

A droplet falling from a certain height possesses an initial velocity *v_0_*, carrying initial kinetic energy *E_0_*. When it contacts the triboelectric surface, it generates both tangential velocity *v*_t_ (parallel to the triboelectric layer) and normal velocity *v*_n_ (perpendicular to the triboelectric interface) (**Figure** **3**). The droplet possesses terminal kinetic energy (*E_1_*) upon separation from the triboelectric layer, and the energy conversion relationships are expressed in Equation 3. In classical researches, when a droplet contacts and slides on an unmodified triboelectric layer, it often fails to separate completely (Figure 3a,I). The pinned droplet hinders the effective release of charge shielding between the droplet and the triboelectric layer, resulting in low *σ_I_* and poor output performance. In this study, the DLP‐modified triboelectric layer effectively prevents droplet pinning (Figure 3a,II), leading to an increase in *σ_I_*. More importantly, compared to unmodified triboelectric layers‐based LS‐TENG (Figure 3a,I), the DLP‐modified triboelectric layer‐based SS‐TENG can operation at a lower tilt angle (*α_1_*) (Figure 3a,II), resulting in a higher *v*_n_ of droplets, thereby generating higher triboelectric energy (*E*_T_). The complete validation process and corresponding experimental results are shown in Figures S7 and S8 (Supporting Information). At the same time, the DLP‐modified slippery triboelectric layer reduced the dissipation energy of droplet (*E*_diss_) – arising from both viscosity resistance at the triboelectric interface and viscous dissipation within the droplet – leading to increased *v*_t_ (Figure 3a,II). This dual effect enhanced charge transfer efficiency while minimizing charge loss energy (*E*_loss_).^[^ ^20^ ^]^ Additionally, the increased *v*_t_ enhances the charge transfer rate in the electrode (*dQ dt^−1^*). The combined improvement in *σ_I_* and *dQ dt* ^−^ *^1^* synergistically enhances the *I*_SC_ and *V*_OC_ of SS‐TENG, as analyzed in Equations 1 and 2.

The energy conversion relationships are expressed as:

(3) $$\begin{array}{c} \Delta E=E_{0}-E_{1}\;=E_{T}+E_{\mathrm{diss}} \end{array}$$

where *ΔE* is the consumed kinetic energy of droplet, The *E*_T_ can be expressed as:

(4) $$E_{T}=E_{\mathrm{out}}+E_{\mathrm{loss}}$$

where *E*_out_ is the output energy of SS‐TENG.

The controllable phase separation preparation of triboelectric layer can be achieved by adjusting the DLP precursor content (Figure S1I, Supporting Information) and lubricant sweating temperature (Figure S1II, Supporting Information) during preparation process. Triboelectric layers with DLP precursor contents ranging from 0 to 7.5 mg cm^−2^ were prepared at a heating temperature of 100 °C. The diameter of the DLPs positively correlated with the precursor content, reaching a saturation size of ≈40 µm at a content of 4.5 mg cm^−2^ (Figure S9, Supporting Information). The output of SS‐TENGs based on these triboelectric layers was tested using a power generation area of 1.5 × 1.5 cm^2^, with deionized water droplets dropped from a height of 10 cm. The *V*_OC_ (Figure 3b) and *I*_SC_ (Figure 3c) of SS‐TENGs reached peak values of 14.4 V and −3.6 µA, respectively, at a precursor content of 4.5 mg cm^−2^ (Figure 3d), representing a significant improvement over the LS‐TENG without DLP modification (0 mg cm^−2^; 0.88 V, −0.06 µA). Triboelectric layers were also prepared at heating temperatures ranging from 80 to 160 °C with a fixed DLP precursor content of 4.5 mg cm^−2^. Higher temperatures increased the activity of the DLP precursor, resulting in a larger diameter and lower density of DLP on triboelectric layer surface (Figure S10, Supporting Information). The *V*_OC_ (Figure 3e) and *I*_SC_ (Figure 3f) of SS‐TENGs reached peak values of 14.4 V and −3.5 µA, respectively, at a heating temperature of 100 °C (Figure 3g). The controllable phase separation preparation method demonstrated that the diameter and density of DLPs can be regulated by adjusting the precursor content and heating temperature. The optimal precursor content and heating temperature for maximizing output performance were determined to be 4.5 mg cm^−2^ and 100 °C, respectively.

#### 2.3.2 Performance of SS‐TENG under Various Operating Conditions

**Figure 4:**
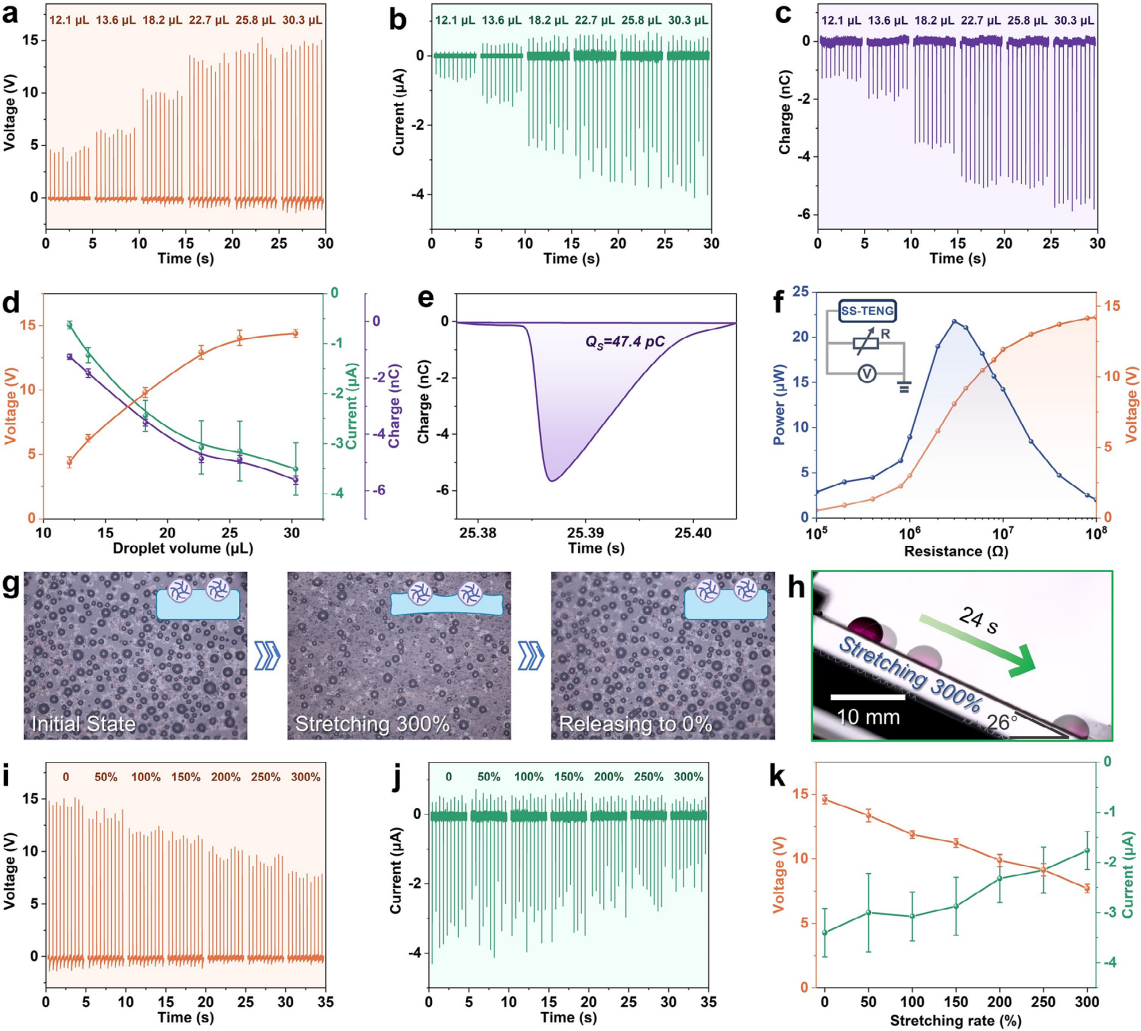
Output performance of SS‐TENG under different conditions. a) *V*_OC_, b) *I*_SC_, and c) *Q*_SC_ at different droplet volumes with power generation area of 1.5 × 1.5 cm^2^ and a drop height of 10 cm. d) The output positively correlated with the droplet volume, reaching a maximum at 30.3 µL. e) Transferred charge by a single droplet. f) Output voltage and power under different resistive loads; inset: test circuit diagram. g) DLPs remain firmly pinned on the triboelectric layer during stretching and releasing. h) The slippery of triboelectric layer is maintained even at a stretching rate of 300%. i) *V*_OC_ and j) *I*_SC_ of SS‐TENG at the stretching rates of 0 –300%, k) with stable performance maintained throughout. Error bars represent the standard deviation of measurements.

The performance of SS‐TENG sample (1.5 × 1.5 cm^2^) was tested under various conditions, with droplets (deionized water) dropped from a height of 10 cm. The influence of droplet size on performance was first analyzed, given the variability of droplet sizes in natural environments. The peak absolute values of *V*_OC_ (**Figure** **4**a), *I*_SC_ (Figure 4b), and short circuit transfer charge (*Q*_SC_) (Figure 4c) showed a positive correlation with droplet volume. As the droplet volume increased from 12.1 to 30.3 µL, *V*_OC_ increased from 4.4 to 14.4 V, *I*_SC_ increased from 0.6 to 3.5 µA, and *Q*_SC_ increased from 1.2 to 5.6 nC (Figure 4d). This is due to the larger droplet translating and inducing more charges with the triboelectric layer, leading to an increase of transfer charge *Q* in the electrode, which further enhance the *V*_OC_ and *I*_SC_, as analyzed in Equations 1 and 2. The *Q* transferred by a single droplet (30.3 µL) was calculated to be 47.4 pC (Figure 4e), determined by integrating the single peak curve in Figure 4c. The *Q* is a key performance metric for LS‐TENGs, as it depends on the droplet's fall height, the area of the LS‐TENG, and the droplet volume. Additionally, the mechanical properties of LS‐TENGs are critical for applications. The SS‐TENG reported in this research exhibits a transferred charge density of 6.95 × 10^−3^ C m^−2^ L^−1^ and exceptional stretchability, outperforming other recent reports (**Table** **1**). The output power (*P*) of SS‐TENG depends on the external load resistance (*R*), as the output voltage (*U*) is positively correlated with resistance (Figure 4f). The *P* was calculated by *U^2^ R^−1^* and reached a maximum of 21.8 µW at an external load resistance of 3 MΩ, corresponding to a power density of 3.2 × 10^3^ W m^−2^ L^−1^. A transistor‐like architecture was incorporated into the SS‐TENG, and *V*_OC_ (Figure S11a, Supporting Information) and *I*_SC_ (Figure S11b, Supporting Information) were systematically characterized before and after this modification. The transistor‐like architecture yielded remarkable performance enhancements, boosting the peak absolute values of *V*_O_ *_C_* to 43.3 V and peak *I*_SC_ to 9.4 µA (Figure S11c, Supporting Information). The peak power output of the transistor‐like architecture‐enhanced SS‐TENG achieved a peak power of 174 µW (Figure S12, Supporting Information). In addition to the comparative parameters presented in Table 1, peak power data have been incorporated in Table S2 (Supporting information).

**Table 1:** Comparison of recent reported LS‐TENG.

	Preparation material	Droplet height [cm]	Transferred charge density [10^−3^ C m^−2^ L^−1^]	Flexibility
[32]	Silicone / ITO glass	35	0.22	No
[35]	PTFE / Ti / Cu	30	3.43	No
[36]	Silicone / Al / ITO glass	30	5.08	No
[37]	PTFE / Cu	30	0.55	Flexible
[38]	BN /PVDF / Al	‐	1.66	Flexible
[25]	PTFE / Silicone / LM	10	4.74	Stretchable
This work	Erucamide / Silicone / LM	10	6.95	Stretchable

Due to the unique preparation process, the DLPs remain firmly pinned on the triboelectric layer surface even when stretched to 300% and released back to the initial state (Figure 4g). The stretch‐release process of the triboelectric layer is demonstrated in Movie S1 (Supporting Information). In the stretched state, the slipperiness of the triboelectric layer slightly decreases due to the reduced DLP density, but it maintains a functional level of slipperiness even at 300% stretching (Figure 4h). The *V*_OC_ (Figure 4i) and *I*_SC_ (Figure 4j) of the SS‐TENG remain stable across stretching rates of 0–300%, with only a minor performance drop caused by reduced slipperiness and increased electrode resistance in the stretched state (Figure 4k). The shear strength of DLPs was evaluated through sandpaper abrasion tests (Figure S13, Supporting information). The interfacial bonding strength of the DLPs was evaluated through cyclic stretch‐bend testing (Figure S14, Supporting information).

#### 2.3.3 Comparative Performance of SS‐TENG and Classical LS‐TENG

**Figure 5:**
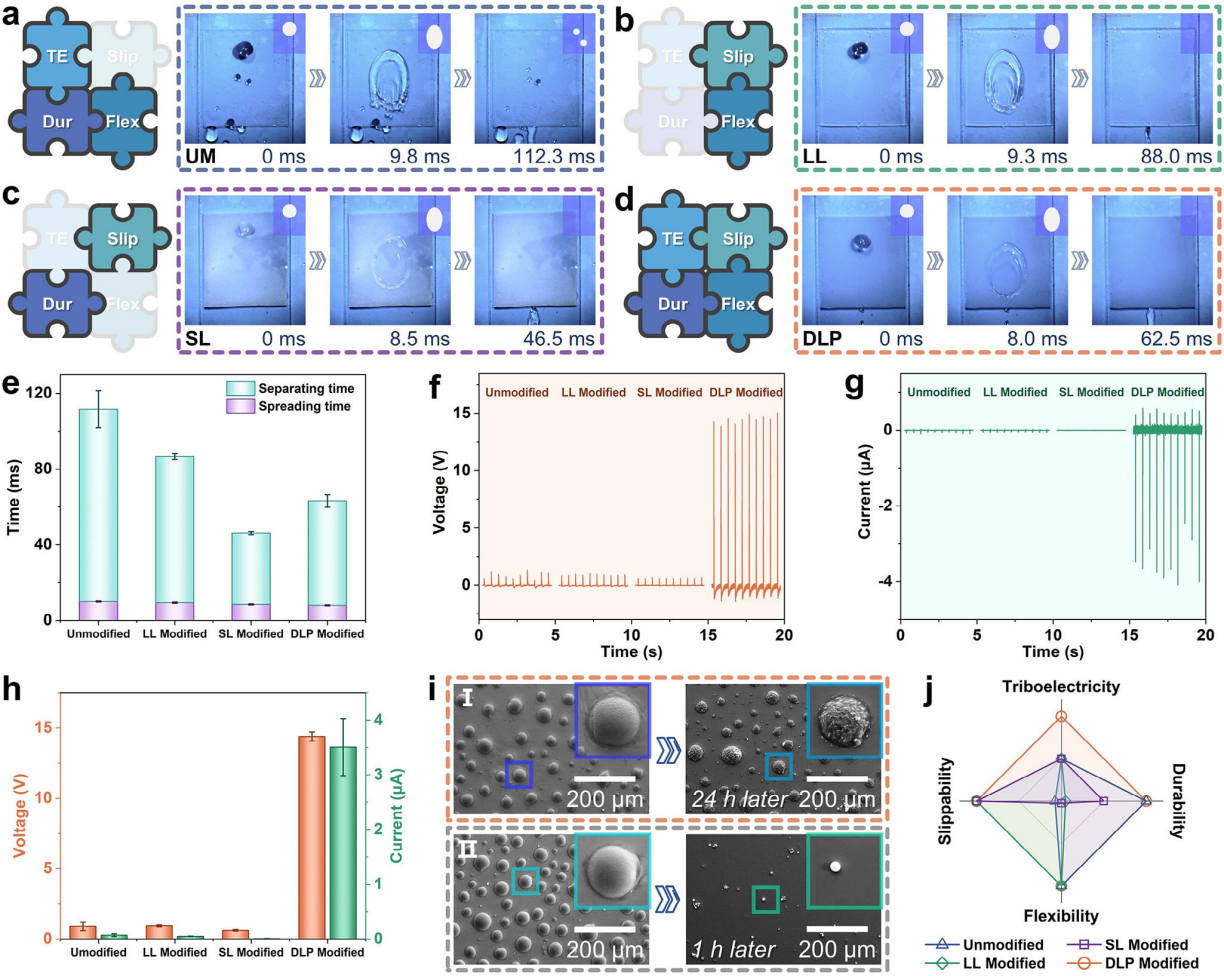
Characterization of LS‐TENGs based on different triboelectric layers, evaluated for triboelectricity (TE), slippability (Slip), durability (Dur), and flexibility (Flex). Droplet behavior on different triboelectric layers with a) unmodified, b) liquid lubricant modified, c) solid lubricant modified, and d) DLP modified. Inset: schematic diagram of droplet interaction with surface. e) Spreading time and separation time of droplets on different triboelectric layers. f) *V*_OC_, g) *I*_SC_ and h) output comparison of LS‐TENGs based on the four types of triboelectric layers. i) SEM images of DLPs on the triboelectric layer after droplet flushing: DLPs with a reinforced skeleton remained intact after 24 h (I), while DLPs without a reinforced skeleton were completely destroyed after 1 h (II). j) Performance distribution graph of LS‐TENGs based on the four types of triboelectric layers. All LS‐TENG samples have a power generation area of 1.5 × 1.5 cm^2^, with droplets (30.3 µL) dropped from a height of 10 cm. Error bars represent the standard deviation of measurements.

To demonstrate the rationality and superiority of SS‐TENG based on the controlled phase separation preparation, the interaction and electrical generation of droplets on different triboelectric layers were compared. Liquid–solid triboelectric nanogenerators were fabricated basing four types of triboelectric layers: unmodified (UM), classical liquid lubricant (LL) modified, classical solid lubricant (SL) modified, and DLP modified (this work). These were evaluated in terms of triboelectricity (TE), slippability (Slip), durability (Dur), and flexibility (Flex). The behavior of droplets on each triboelectric layer was captured using a high‐speed camera and is presented in Movie S2 (Supporting Information). The UM triboelectric layer was prepared using pure silicone without any additional materials. Due to the absence of lubricant on the UM triboelectric layer surface, droplet partially pinned to the surface after contact and separation (**Figure** **5**a). The classical LL modified triboelectric layer was prepared by spraying silicone oil onto silicone, followed by vacuuming at −0.09 MPa for 30 min and spin‐coating at 1000 rpm for 30 s to remove excess silicone oil. Droplets slipped faster and separated completely from the LL modified triboelectric layer (Figure 5b). However, the silicone oil blocked direct contact between the droplets and the triboelectric layer, negatively impacting output performance. Additionally, the durability of the LL modified triboelectric layer was poor, as the silicone oil was washed away by droplets after several cycles. The classical SL modified triboelectric layer was prepared by replacing erucamide with paraffin wax, following the same process as described in Figure S1 (Supporting Information). Unlike the dispersed particles in the DLP‐modified triboelectric layer, a continuous wax film formed on the triboelectric layer (Figure S15I, Supporting Information). Droplets also slipped faster and separated completely from the SL modified triboelectric layer (Figure 5c). While the SL modified layer retained normal durability, it lacked stretchability, as the solid lubricant surface tended to crack and peel off after repeated stretching (Figure S15II, Supporting Information). In contrast, the DLP modified triboelectric layer, prepared using the controllable phase separation method in this research, featured uniformly dispersed lubricant particles on its surface. This design enhanced slipperiness while ensuring direct contact between droplets and the triboelectric layer (Figure 5d). The spreading time (from contact to spreading) and separation time (from spreading to separation) of droplets on different triboelectric layers are shown in Figure 5e, demonstrating that lubricants significantly improve droplet separation efficiency.

The *V*_OC_ (Figure 5f) and *I*_SC_ (Figure 5g) of LS‐TENGs based on the above different triboelectric layers were measured. The peak values of *V*_OC_ for the four types of LS‐TENGs are 0.89, 0.93, 0.63, and 14.39 V, while the peak values of *I*_SC_ are −0.07, −0.05, −0.009, and −3.51 µA, respectively (Figure 5h). The output of the SS‐TENG (DLP modified) was significantly higher than that of other samples, as the lubricant particles enhanced the droplet slip rate and separation efficiency while ensuring effective interfacial charge transfer between the droplet and the triboelectric layer. Furthermore, the DLP modified triboelectric layer, prepared using the controllable phase separation method, exhibits excellent scouring resistance due to the reinforced skeleton, in addition to its stretchability. Even after 24 h of droplet washing at a height of 10 cm and a frequency of 2 Hz, the lubricant particles on the DLP‐modified triboelectric layer showed no significant damage (Figure 5i,I). In contrast, lubricant particles without a reinforced skeleton were almost entirely destroyed under the same conditions after just 1 h (Figure 5i,II). The output performance of DLP (without skeleton) modified SS‐TENG was systematically evaluated over 0–50 min of operation at 10 min intervals (Figure S16, Supporting Information). In summary, the DLP modified triboelectric layer demonstrates unique superiority over other triboelectric layers, combining triboelectricity (TE), slippability (Slip), durability (Dur), and flexibility (Flex), as illustrated in Figure 5j.

### 2.4 Application Demonstration of SS‐TENG in Multiple Scenarios

**Figure 6:**
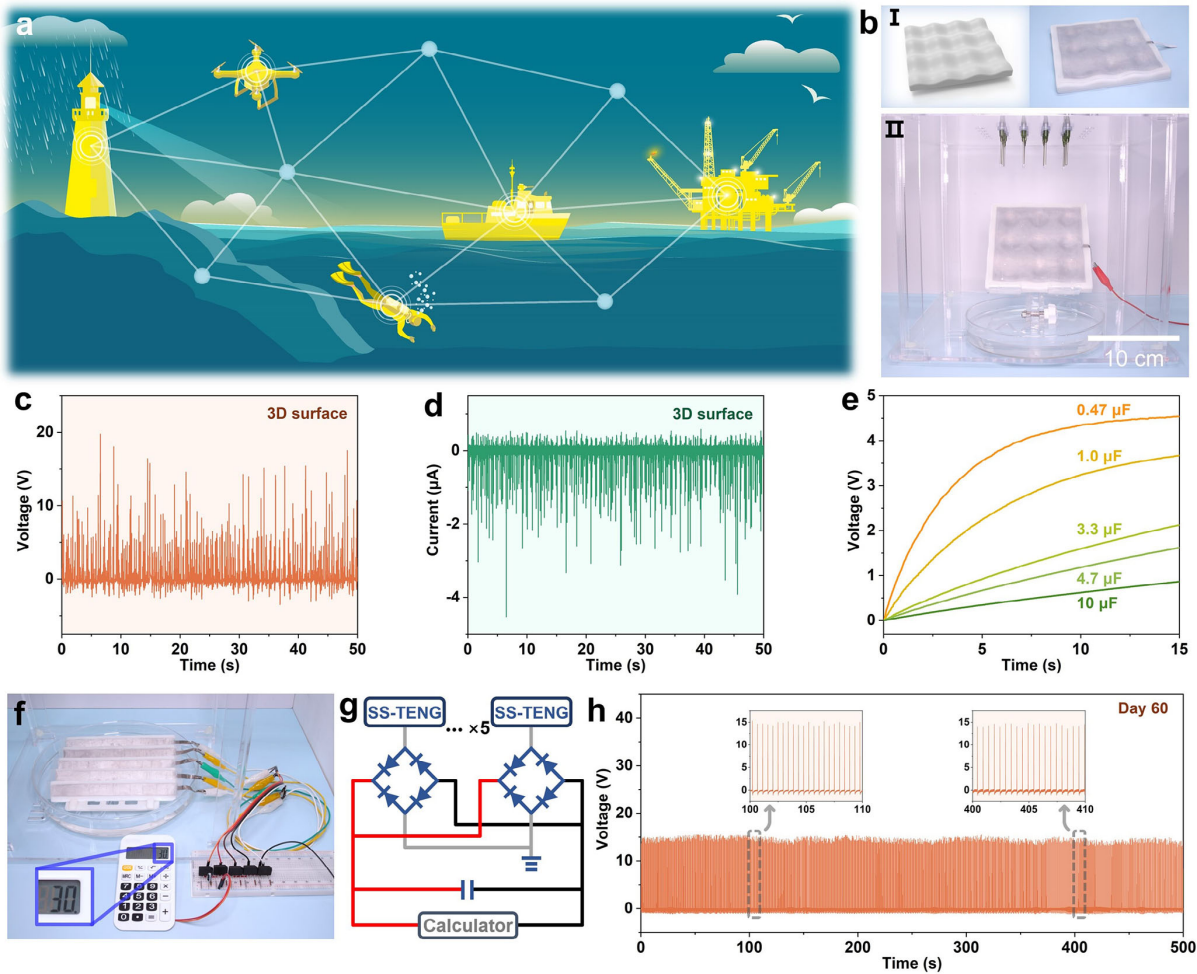
Application demonstration of SS‐TENG. a) Potential applications of the SS‐TENG, illustrating its integration with various objects to harvest low‐intensity water energy and power IoT devices. b) A SS‐TENG sample (10 cm × 10 cm) fitted on a 3D surface (I) to harvest droplet energy (II). c) *V*_OC_ and d) *I*_SC_ of the SS‐TENG under multiple droplets. e) Voltage charging curves for different capacitors. f) Five SS‐TENG samples (150 mm × 15 mm) placed in a simulated rainy environment to harvest droplet energy and power a calculator. Inset: The calculator functioning properly. The flow rate is 50 mL min^−1^. g) Schematic diagram of the experimental circuit. h) Long‐term stability test of the SS‐TENG, showing *V*_OC_ after 2 months. Inset: *V_OC_* at 100 and 400 s, demonstrating no decline in performance.

With these advantages, the proposed SS‐TENG can be seamlessly integrated into buildings, vehicles, and clothing to harvest low‐intensity water energy and power IoT devices, as illustrated in **Figure** **6**a. The SS‐TENG sample demonstrated excellent adaptability, fitting tightly on flat surfaces (Figure S17aI, Supporting Information), curved surfaces (Figure S18aI, Supporting information), and 3D surfaces (Figure 6b,I). The output performance of the SS‐TENG was measured on these surfaces under multiple droplets (4 × 3) with a water flow rate of 26 mL min^−1^. No significant decline in *V*_OC_ or *I*_SC_ was observed when the SS‐TENG was fitted on a 3D surface (Figure 6c,d) or a curved surface (Figure S18b,c, Supporting Information), compared to its performance on a flat surface (Figure S17b,c, Supporting information), confirming stable output even under deformation. The capacitor charging and device‐driving capabilities of the SS‐TENG were also evaluated. Five SS‐TENG samples (150 mm × 15 mm) were placed in a simulated rainy environment with a flow rate of 50 mL min^−1^ (Figure S19, Supporting information). After rectifying the AC output of the SS‐TENGs to DC, various capacitors (0.47, 1.0, 3.3, 4.7, 10 µF) were charged (Figure 6e). The 0.47 µF capacitor reached 4.5 V in 15 s, demonstrating sufficient energy to power most low‐power devices. A miniature calculator, connected in parallel with the capacitor, was successfully powered by the SS‐TENG and operated in real time, as shown in Movie S3 (Supporting Information) and Figure 6f. The external circuit diagram is illustrated in Figure 6g. Long‐term stability tests were conducted by measuring *V*_OC_ over 500 s at one‐month interval (Figure S20a,b, Supporting Information, and Figure 6h). *V*_OC_ showed almost no decline after two months, highlighting the excellent long‐term stability of the SS‐TENG. The detail comparison of the *V*_OC_ and water contact angles of the triboelectric layer at operation time of 100 and 400 s (Figure 6h) was presented in Figure S21 (Supporting Information).

## 3 Conclusion

In summary, a stretchable slippery triboelectric nanogenerator was proposed in this research, in which the DLP on the triboelectric layer were prepared using a controllable phase separation method. The DLPs preserved the triboelectric effect between droplets and the triboelectric layer while enhancing lubricity. The improved lubricity increased the efficiency of charge shield release, resulting in a *V*_OC_ of 14.4 V for the SS‐TENG, over 15 times higher than that of the LS‐TENG with an unmodified triboelectric layer. The durability of the DLPs was significantly improved by the epoxy resin skeleton, allowing them to remain intact on the triboelectric layer even after 24 h of droplet washing. The *V*_OC_ showed almost no decline after 2 months or over 1000 cycles of operation. The DLPs remained firmly bonded to the triboelectric layer even under bending or stretching, ensuring exceptional flexibility compared to classical solid lubricant surfaces. The SS‐TENG maintained stable output even at a stretching rate of 300%. In a practical demonstration, an SS‐TENG with an area of 112.5 cm^2^ successfully powered a calculator under a droplet flow rate of 50 mL min^−1^. With its superior electrical and mechanical properties, the SS‐TENG can be integrated into various objects to harvest low‐intensity water energy and power IoT devices.

## 4 Experimental Section

### Materials for Preparation

Erucamide, Epoxide resin precursor E51 (E51‐EP), Poly(propylene glycol) bis(2‐aminopropyl ether) (PEA), dimethylsilicone oil (viscosity 100 mPa.s), paraffin wax (melting point: 58–60 °C) were purchased from MACKLIN. Silicone (Ecoflex 00–30) was purchased from Smooth‐On, Inc. Liquid metal (LM, 68.5% Ga, 21.5% In, and 10% Sn) were purchased from Shanghai Aladdin Biochemical Technology Co., Ltd.

### Preparation of SS‐TENG

The triboelectric layer was fabricated through a controllable phase separation process, consisting of Ecoflex as the triboelectric material, erucamide as the lubricating microparticles, and epoxy resin forming the internal skeleton of the lubricating particles. The electrode layer comprises liquid metal, while the substrate is made of Ecoflex, enabling seamless integration with the triboelectric layer and effective encapsulation of the electrode layer. The detailed preparation process of SS‐TENG is provided in the Supporting Information.

### Characterization of SS‐TENGs

The surface structure was characterized by focused ion beam scanning electron microscope (SEM, Helios G4 CX, Czech), energy dispersive spectroscopy (EDS), and optical microscope (BX51, Olympus, Japan). The surface ingredient was characterized by Fourier transform infrared (FTIR) spectroscopy (PerkinElmer, Frontier, American) The capacitance of the triboelectric layer was measured by impedance analyzer (Keysight E4990A, USA) over a frequency range of 1 kHz to 10 MHz. The tensile property was measured by electromechanical universal testing machine (Wance 102 A, China). The voltage, current and transferred charge were measured by electrostatic meter (Keithley 6514, USA). The behavior of droplets on the triboelectric layer was captured by high‐speed camera (FASTCAM Nova S9, Photron, Japan).

## Conflict of Interest

The authors declare no conflict of interest.

## Supporting information



Supporting Information

Supplemental Movie 1

Supplemental Movie 2

Supplemental Movie 3

## Data Availability

The data that support the findings of this study are available from the corresponding author upon reasonable request.
