# Supplementary material for: Skeleton Enhanced Dispersed Lubricant Particle Based Triboelectric Nanogenerator for Droplet Energy Harvesting
Source: Adv Sci (Weinh). 2025 May 28;12(31):e05363. doi: 10.1002/advs.202505363 (PMC12376673; doi:10.1002/advs.202505363)
Supplement: Supplementary file 1 — Supporting Information [file ADVS-12-e05363-s001.docx]

Supporting Information

**Skeleton enhanced dispersed lubricant particle based triboelectric nanogenerator for droplet energy harvesting**

*Changjun Yang, Yan Wang, Yamei Wang, Zelinlan Wang, Yurun Guo, Liwen Zhang, Xiaolin Liu*, Huawei Chen**

C. Yang, Y. Wang, Z. Wang, Y. Guo, Prof. L. Zhang, Prof. X. Liu, Prof. H. Chen

School of Mechanical Engineering and Automation

Beihang University

Beijing 100191, China

E-mail: chenhw75@buaa.edu.cn

Y. Wang

School of Mechanical Engineering

Dalian University of Technology

Dalian 116024, China

Prof. H. Chen

Beijing Advanced Innovation Center for Biomedical Engineering

Beihang University

Beijing 100191, China

**Detailed derivation process for SS-TENG output.**

The droplet triboelectric power generation in this study utilizes the surface electrostatic charge generated by the contact of droplets and triboelectric layer, which creates a time-varying electric field to drive electrons flow in the external circuit. The electrostatic field established by the triboelectric charge drives the electrons flow through the external load, resulting in the free charge *σ_I_(z, t)* accumulating in electrode. The generation mechanism can be derived from the capacitance model, where the electrode possesses a potential of *V* and the capacitance changes over time leading to the transfer charge *Q* in electrode, which creates a short circuit current (*I_SC_*) in the external circuit:

$\begin{matrix} I_{SC}=\frac{dQ}{dt}=A\frac{d\sigma_{I}\left(z,t\right)}{dt} \\ =C\frac{dV}{dt}+V\frac{dC}{dt} \end{matrix}$ (S1)

The relationship between the voltage in external circuit and open circuit voltage *V_OC_* is given by

$RI=V_{OC}-Q/C$ (S2)

$RA\frac{d\sigma_{I}\left(z,t\right)}{dt}=V_{OC}-A\sigma_{I}\left(z,t\right)/C$ (S3)

Therefore, the *V_OC_* can be stated as

$\begin{matrix} V_{OC}=RA\frac{d\sigma_{I}\left(z,t\right)}{dt}+A\frac{\sigma_{I}\left(z,t\right)}{C} \\ =R\left(C\frac{dV}{dt}+V\frac{dC}{dt}\right)+A\frac{\sigma_{I}\left(z,t\right)}{C} \end{matrix}$ (S4)

**Table S1.** The meaning of each notation in Equation S1-S4.

| Notation | Meaning |
| --- | --- |
| *I_SC_* | short circuit current |
| *V_OC_* | open circuit voltage |
| *Q* | transfer charge |
| *t* | contact-separation time |
| *A* | droplet spreading area |
| *σ_I_* | free charge density in electrode |
| *z* | distance between droplet and triboelectric layer |
| *C* | capacitance |
| *V* | voltage in external circuit |
| *R* | external load resistance |
| *I* | current in external circuit |


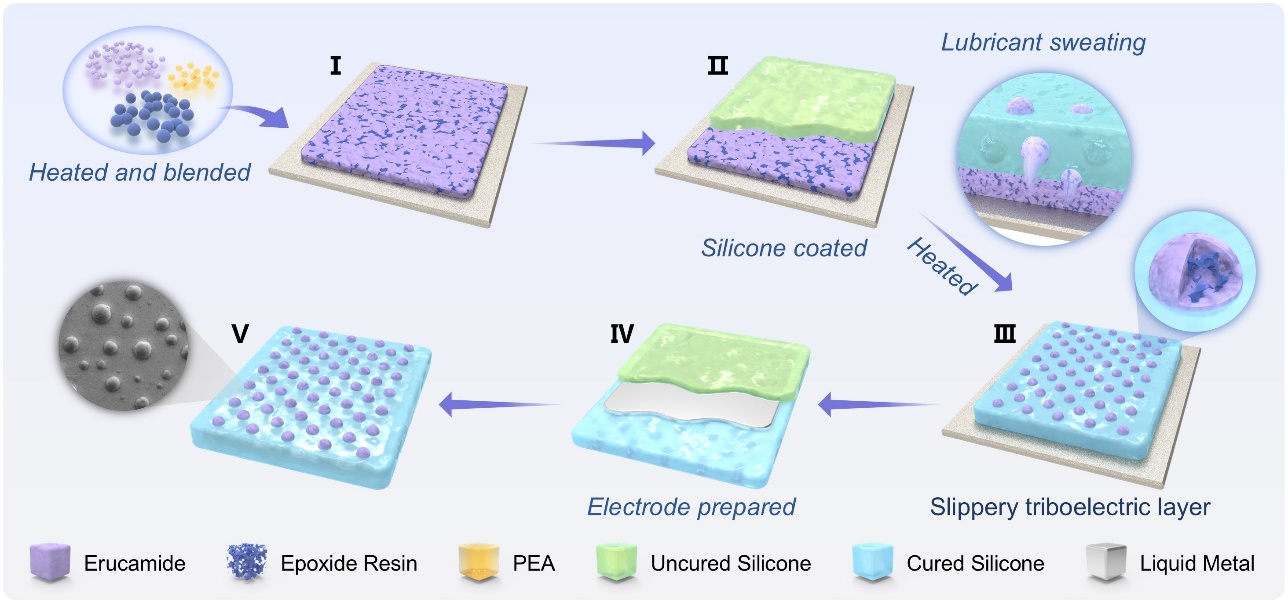


**Figure S1.** Preparation process of SS-TENG. Erucamide, E51-EP and PEA are dissolved and blended to prepare the precursor of skeleton enhanced lubricant particle, and the precursor was applied to a plate (I); Silicone mixture is coated on the precursor (II), and the plate is heated to prepare the dispersed lubricant particle based slippery triboelectric layer (III). Inset: schematic diagram of skeleton in the lubricant particle; Electrode is prepared on the back side of triboelectric layer and sealed by the silicone (IV); SS-TENG is prepared completely after the silicone cured (V). Inset: SEM image of triboelectric layer interface, showing lubricant particles bond on the triboelectric layer.

*Preparation of SS-TENG:* To prepare the precursor of skeleton enhanced lubricant particle, erucamide and E51-EP (mass ratio of 2:1) were fully dissolved and blended at 85 °C (Figure S1, Supporting information). PEA (mass ratio of 1:3 to E51-EP) was then added to the mixture. The precursor was applied to a plate with a weight of 4.5 mg cm^−2^ (Figure S1-I, Supporting information). Two parts of Ecoflex 00-30 silicone (mass ratio of 1:1) were mixed and coated onto the precursor layer using an adjustable film applicator (Figure S1-II, Supporting information). The plate was heated to 100 °C to sweat erucamide and E51 onto the silicone surface while curing the silicone. After 3 hours of heating, dispersed lubricant particles (DLPs) formed on the cured silicone, with erucamide as the lubricant component and E51 as the skeleton component (Figure S1-III, Supporting information). LM was deposited on the backside of the triboelectric layer to prepare the electrode film, which was then sealed with Ecoflex 00-30 silicone (Figure S1-IV, Supporting information). The SS-TENG was fully prepared after curing for 4 hours at 25 °C (Figure S1-V, Supporting information). Inset scanning electron microscope (SEM) images show the lubricant particles strongly bonded to the triboelectric layer.


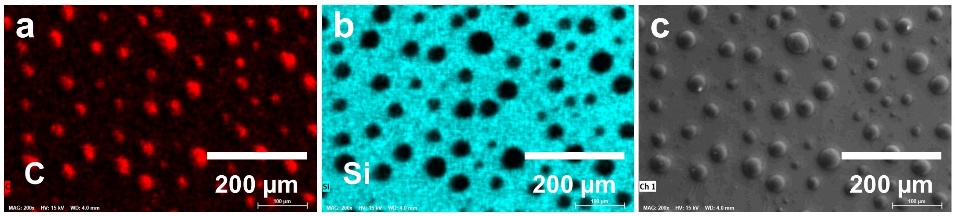


**Figure S2.** EDS images of elemental a) C and b) Si, related to c) the SEM image of triboelectric layer interface.


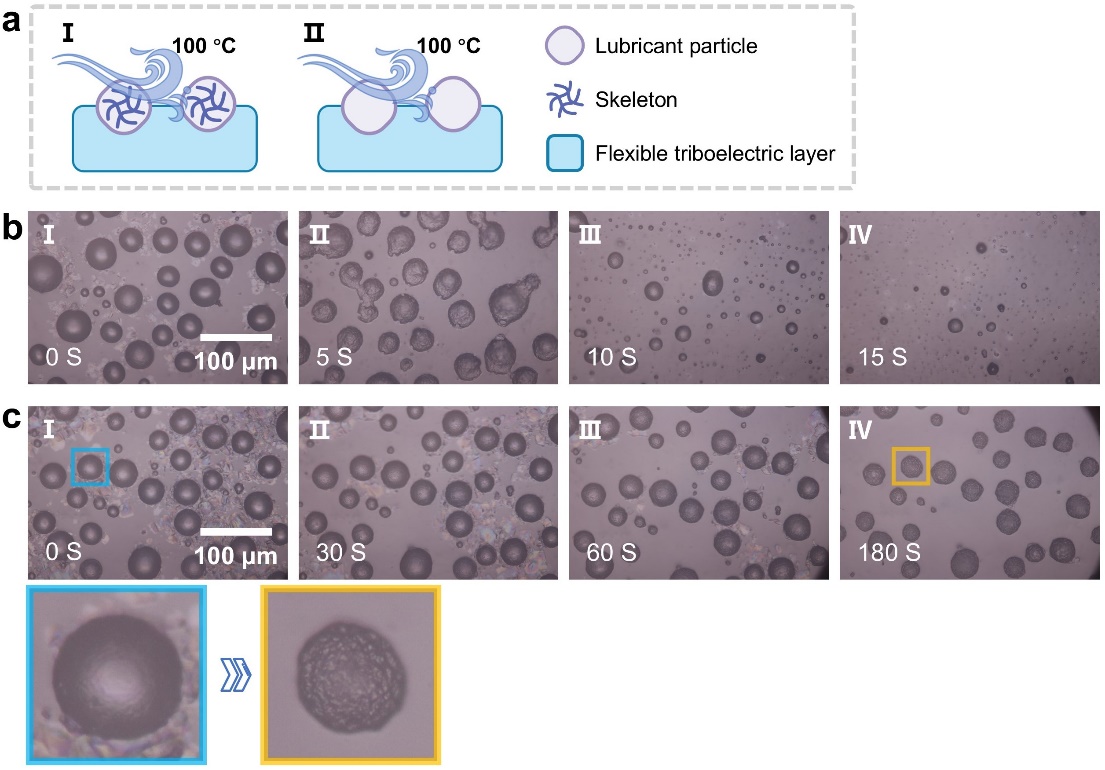


**Figure S3.** a) Schematic diagram of hydrothermal flushing test. b) The skeleton-free DLPs exhibited complete dissolution upon water exposure. c) The skeleton-incorporated DLPs demonstrated progressive surface erucamide dissolution, ultimately revealing their embedded epoxy resin skeleton.

Two distinct types of slippery triboelectric layers were fabricated, differentiated by the presence or absence of reinforced skeleton within the dispersed lubricant particles (DLPs). Hydrothermal flushing tests were conducted on both samples using 100 °C water, higher than the melting point of erucamide (79 °C ~ 81 °C), at a flow rate of 0.1 mL/s (Figure S3a). The skeleton-free DLPs underwent rapid dissolution upon water exposure (Figure S3b), with near-complete disappearance observed after 15 s. In contrast, the skeleton-incorporated dispersed lubricant particles (DLPs) exhibited significantly reduced dissolution rates under hydrothermal flushing. Remarkably, these reinforced particles maintained structural integrity even after 180 s of exposure, with complete dissolution of surface erucamide components revealing the underlying epoxy resin skeleton (Figure S3c).


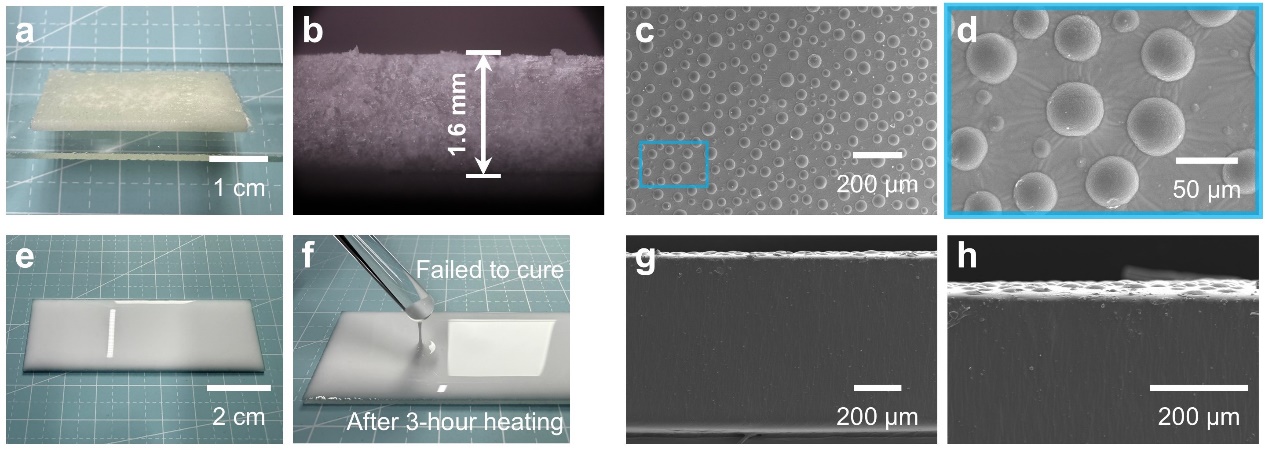


**Figure S4.** a) Digital photograph and b) cross-sectional micrograph of the erucamide-epoxy composite. c, d) SEM images of DLP without the epoxy resin skeleton on the triboelectric layer. e) Digital photograph of the silicone-epoxy mixture, and f) the mixture failed to cure after being heated at 100 °C for 3 hours. SEM images of triboelectric layer showing g) cross-sectional morphology and h) magnified cross sectional details.

Three experiments were conducted to further explain the mechanism of the controllable phase separation.

*Experiment 1:* Erucamide and E51-EP (mass ratio of 2:1) were fully dissolved and blended at 85 °C. PEA (mass ratio of 1:3 to E51-EP) was then added to the mixture, and heated to 100 °C to cured (Figure S4a). The erucamide and the E51-EP were fully blended without exhibiting stratification (Figure S4b).

*Experiment 2:* In the absence of epoxy resin, phase separation between erucamide and silicone was achieved using the same preparation method as described in the Experimental section, resulting in the dispersed lubricant particle (DLP) without the epoxy resin skeleton on the triboelectric layer surface (Figure S4c and S4d).

*Experiment 3:* Silicone and E51-EP (mass ratio of 10:1, PEA (mass ratio of 1:3 to E51-EP)) were fully mixed and coated on glass substrate (Figure S4e). The coating silicone-epoxy mixture failed to cure after being heated at 100 °C for 3 hours (Figure S4f).

In summary, erucamide can fully blend with epoxy (Experiment 1), and achieve phase separation with silicone rubber (Experiment 2), while epoxy resin remains incompatible with silicone (Experiment 3). Therefore, the proposed controllable phase separation method enables epoxy-loaded erucamide to phase-separate from silicone precursor under the heating condition, followed by successful curing.

The phase separation method developed in this study achieves complete segregation between the erucamide/epoxy resin composite and the silicone rubber, forming lubricant particles with an internal skeleton. As evidenced in Figure S4g and S4h, no residual erucamide/epoxy resin remains within the triboelectric layer, confirming thorough phase separation.


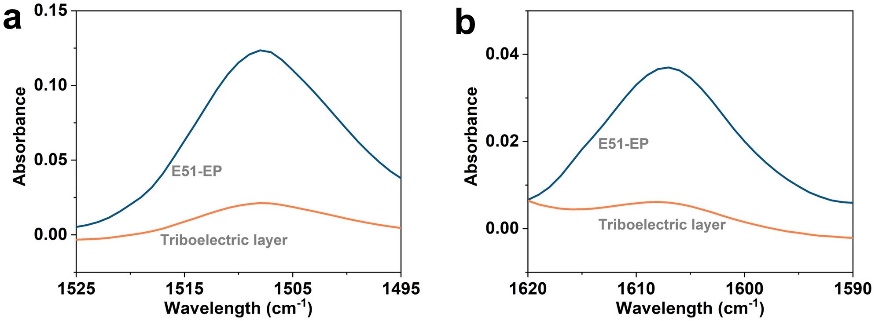


**Figure S5.** Local enlarged view for vibration peaks at a) 1509 and b) 1607 cm^−1^.


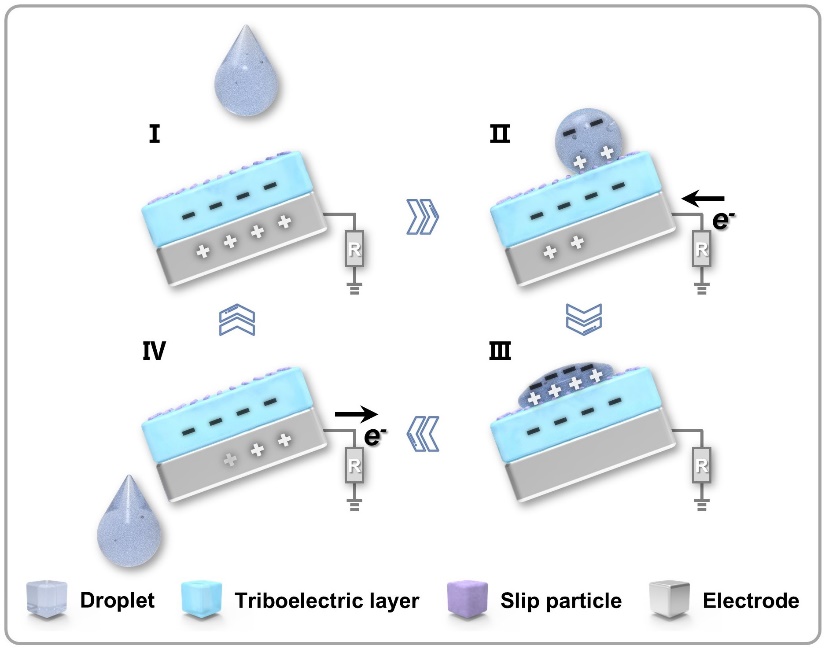


**Figure S6.** The working principle of SS-TENG.

The working principle of SS-TENG is illustrated in **Figure S6**. Initially, when a droplet comes into contact with the triboelectric layer, charge transfer occurs at the liquid-solid contact interface. After several repeated droplet contacts, the triboelectric layer accumulates more negative charges until saturated. At the separate state (**Figure S6-i**), the triboelectric layer induces an equal amount of positive charge in the electrode layer, establishing an electrostatic equilibrium. When the droplet contacts with the triboelectric layer (**Figure S6-ii**), charge shielding occurs between their surfaces, breaking the electrostatic equilibrium. Electrons continuously flow from the ground to the electrode layer until the droplet spreads sufficiently on the triboelectric layer (**Figure S6-iii**). Subsequently, the charge shielding fades as droplet sliding off the triboelectric layer, and electrostatic equilibrium re-occurs. Electrons continuously flow from the electrode layer to the ground during this process (**Figure S6-iv**). In summary, the process of a droplet going from contact to separate state generates an alternating current, and electrical energy is continuously generated as droplets continue to fall.


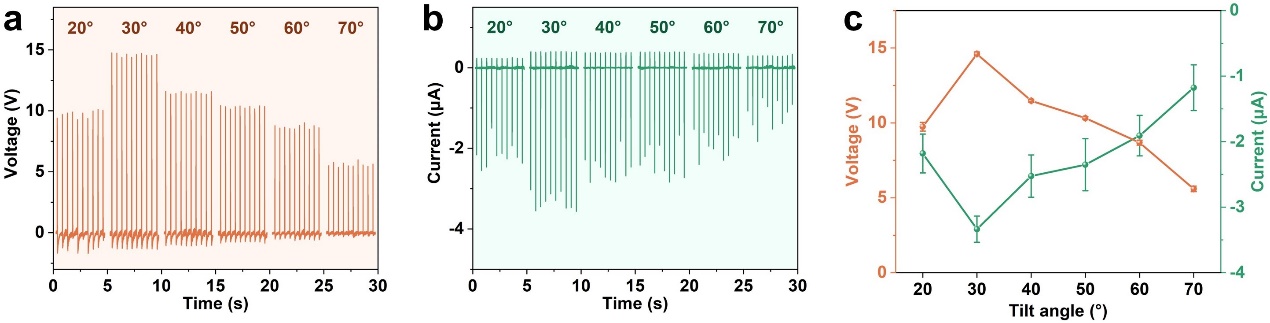


**Figure S7.** Output of the SS-TENG under different tilt angles. a) *V_OC_* and b) *I_SC_* under different tilt angles, c) reaching peak values at a tilt angle of 30°.

The slippery triboelectric layer enables SS-TENG operation at lower tilt angles, thereby increasing mechanical energy input of droplets. According to electron cloud theory governing charge transfer at solid-liquid interfaces (*Droplet 2024, 3(1), e97*), this enhanced mechanical energy input directly correlates with improved charge transfer capacity across the interface. To validate this conclusion, the output performance of both the SS-TENG and a liquid-solid TENG (LS-TENG) based on pristine Ecoflex polymer was systematically characterized across varying tilt angles (20° ~ 70°). While lower tilt angles of the SS-TENG facilitate higher droplet mechanical energy, they hinder droplet detachment. The DLP-modified SS-TENG achieves effective droplet separation at 30° tilt angle, delivering peak outputs of 14.6 V (*V_OC_*) (**Figure S7a**) and -3.3 μA (*I_SC_*) (**Figure S7b**). Although increased angles accelerate droplet movement, the reduced mechanical energy input leads to deteriorating output performance (**Figure S7c**). In contrast, for the liquid-solid TENG (LS-TENG) based on pristine Ecoflex polymer, droplet pinning occurs after contact-separation. This pinning effect weakens with increasing tilt angles, enhancing electrical outputs to maximum values of 0.9 V (*V_OC_*) (**Figure S8a**) and -0.06 μA (*I_SC_*) (**Figure S8b**) at 60°. However, excessive tilt (70°) causes insufficient mechanical energy input, resulting in performance decline (**Figure S8c**).


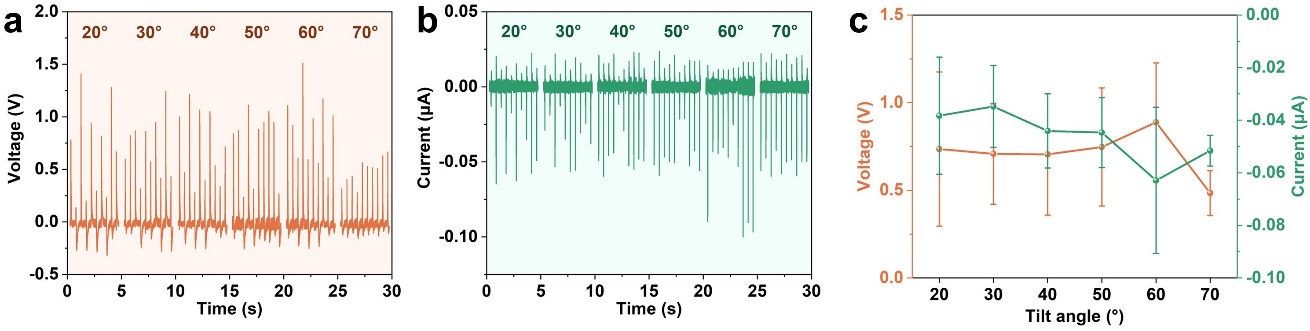


**Figure S8.** Output of a LS-TENG based on pristine Ecoflex polymer under different tilt angles. a) *V_OC_* and b) *I_SC_* under different tilt angles, c) reaching peak values at a tilt angle of 60°.


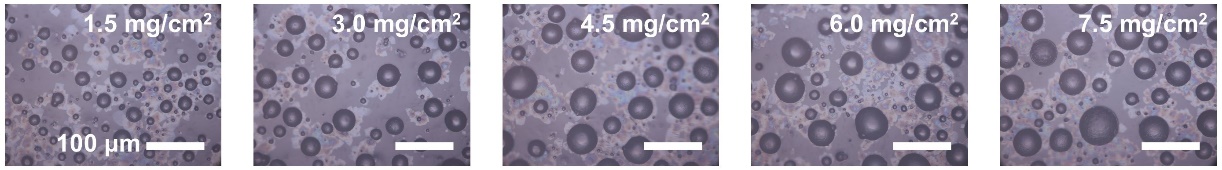


**Figure S9.** Magnify images of triboelectric layer interface under different DLP precursor contents (1.5 ~ 7.5 mg cm^−2^) at the heating temperature of 100 ℃.


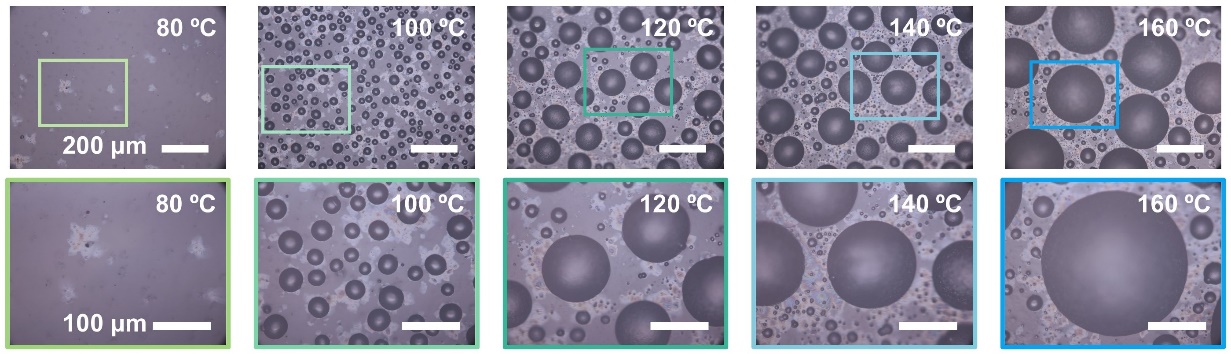


**Figure S10.** Magnify images of triboelectric layer interface at different heating temperatures (80 ~ 160 ℃) with the DLP precursor content of 4.5 mg cm^−2^.


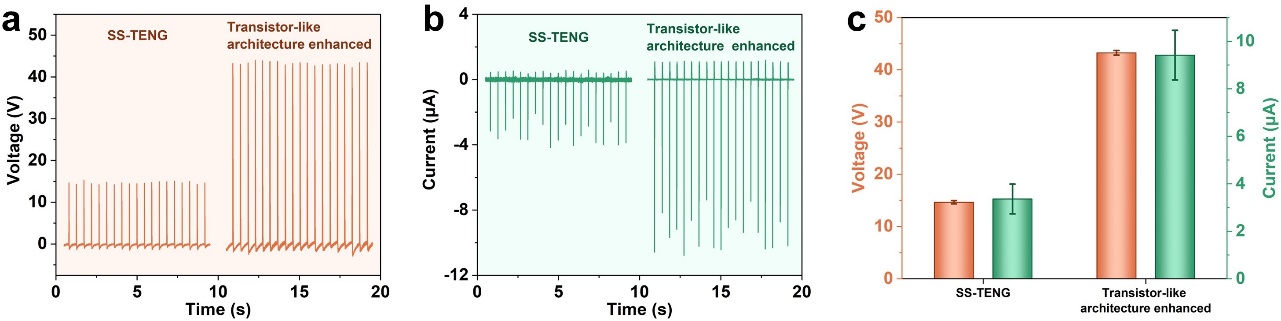


**Figure S11.** Output of SS-TENGs before and after the transistor-like architecture modification. a) *V_OC_*, b) *I_SC_* and h) output comparison of SS-TENGs.

A transistor-like architecture was incorporated into the SS-TENG, and *V_OC_* (Figure S11a) and *I_SC_* (Figure S11b) were systematically characterized before and after this modification. The transistor-like architecture yielded remarkable performance enhancements, boosting the peak absolute values of *V_OC_* from 14.6 V to 43.3 V and peak *I_SC_* from 3.4 μA to 9.4 μA (Figure S11c).


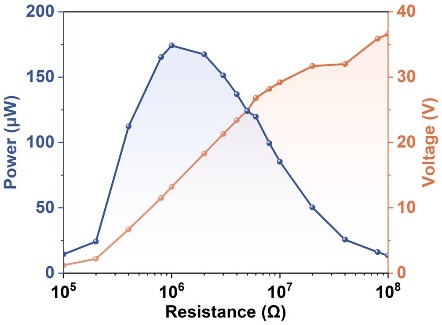


**Figure S12.** Output voltage and power of the transistor-like architecture-enhanced SS-TENG under different resistive loads.

The peak power output of the transistor-like architecture-enhanced SS-TENG was also evaluated. At an external load resistance of 1 MΩ, the device achieved a peak power of 174 μW (Figure S12), corresponding to a peak power density of 25.5 kW m^−2^ L^−1^. It should be noted that these power values represent instantaneous measurements and may not fully characterize the SS-TENG’s sustained output performance.

**Table S2.** Comparison of recent reported LS-TENG with peak power density.

|  | Preparation material | Droplet height (cm) | Transferred charge density (10^−3^ C m^−2^ L^−1^) | Peak power density (kW m^−2^ L^−1^) | Flexibility |
| --- | --- | --- | --- | --- | --- |
| ^[32]^ | Silicone / ITO glass | 35 | 0.22 | - | No |
| ^[35]^ | PTFE / Ti / Cu | 30 | 3.43 | - | No |
| ^[36]^ | Silicone / Al / ITO glass | 30 | 5.08 | 9.37* | No |
| ^[21]^ | PTFE / ITO /Al | 15 | - | 501* | No |
| ^[37]^ | PTFE / Cu | 30 | 0.55 | - | Flexible |
| ^[38]^ | BN /PVDF / Al | - | 1.66 | - | Flexible |
| ^[25]^ | PTFE / Silicone / LM | 10 | 4.74 | 0.0069 | Stretchable |
| This work | Erucamide / Silicone / LM | 10 | 6.95 | 3.2 | Stretchable |
| This work | Erucamide / Silicone / LM / Al | 10 | - | 25.52* | Stretchable |

*Note: With transistor-like architecture.

Based on the existing comparison items in Table 1, authors have additionally included peak power comparison and incorporated relevant reported literature as shown in Table S2, with appropriate citations to the related references. These descriptions have been stressed in the manuscript and supporting information.


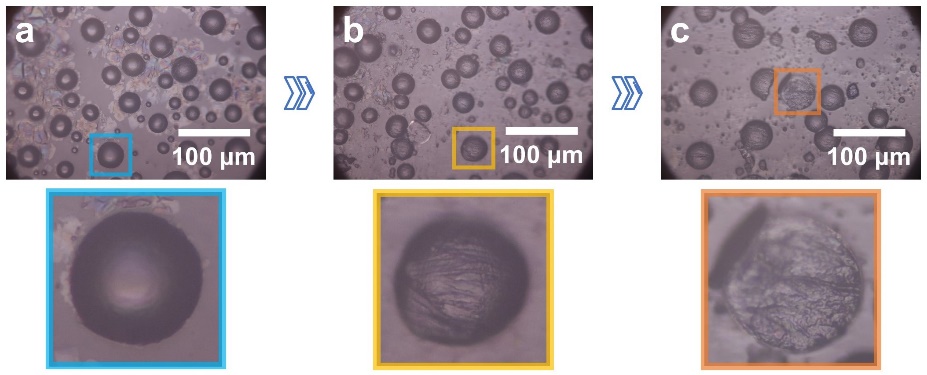


**Figure S13.** The shear strength test of DLP. DLPs on the triboelectric later a) at initial state, b) after sandpaper abrasion under a 1 N normal force and c) under a 5 N normal force.

The shear strength of DLPs was evaluated through sandpaper abrasion tests, where 600-grit sandpaper was placed on the triboelectric layer surface and dragged for 1 m under different normal force. Compared to the initial smooth surface (Figure S13a), the DLP surface showed scratching marks under a 1 N normal force, but maintained structural integrity without damage (Figure S13b). When the normal force was increased to 5 N, partial damage occurred in the DLPs, exhibiting fracture phenomena (Figure S13c). These sandpaper abrasion tests conclusively demonstrate the superior shear strength of DLPs.


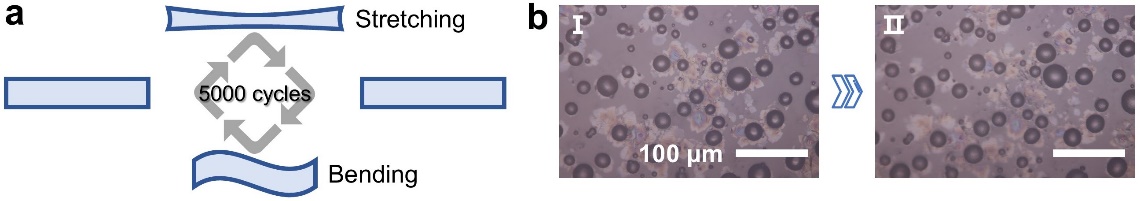


**Figure S14.** The interfacial bonding strength test of DLP. a) Schematic diagram of cyclic stretch-bend testing. b) DLPs on the triboelectric later at initial state (I) and after 5000 stretching-bending cycles (II).

The interfacial bonding strength of the DLPs was evaluated through cyclic stretch-bend testing (Figure S14a). After 5000 stretching-bending cycles, the DLPs remained firmly bonded at the triboelectric layer interface with no observed delamination (Figure S14b). The cyclic stretch-bend test conclusively demonstrates the superior interfacial bonding strength of DLPs. These descriptions have been stressed in the manuscript and added in the supporting information.


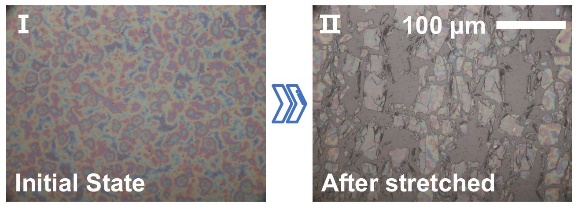


**Figure S15.** The classical solid lubricant (I) on the triboelectric layer tends to break and peel (II) after stretch-release.


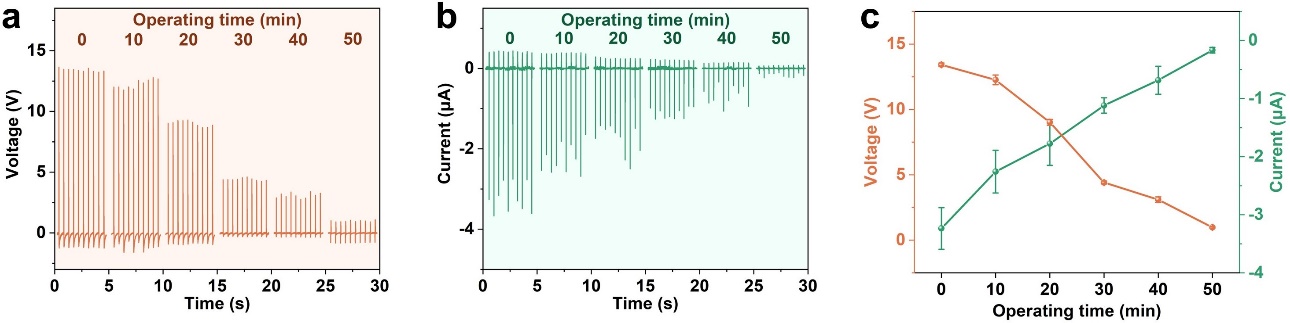


**Figure S16.** a) *V_OC_* and b) *I_SC_* of DLP (without skeleton) modified SS-TENG over 0 ~ 50 minutes of operation at 10-minute intervals. c) The peak *V_OC_* and *I_SC_* significantly decreased from 0 min to 50 min of operation. Error bars represent the standard deviation of measurements.

The output performance of DLP (without skeleton) modified SS-TENG was systematically evaluated over 0 ~ 50 minutes of operation at 10-minute intervals (Figure S16a and S16b). Without the embedded skeleton, the surface DLP suffered damage during droplet impact, leading to deteriorated slippery performance and consequently reduced electrical output. The peak absolute values of *V_OC_* and *I_SC_* significantly decreased from initial values of 13.4 V and 3.3 μA at 0 min to merely 1.0 V and 0.2 μA after 50 min of operation (Figure S16c).


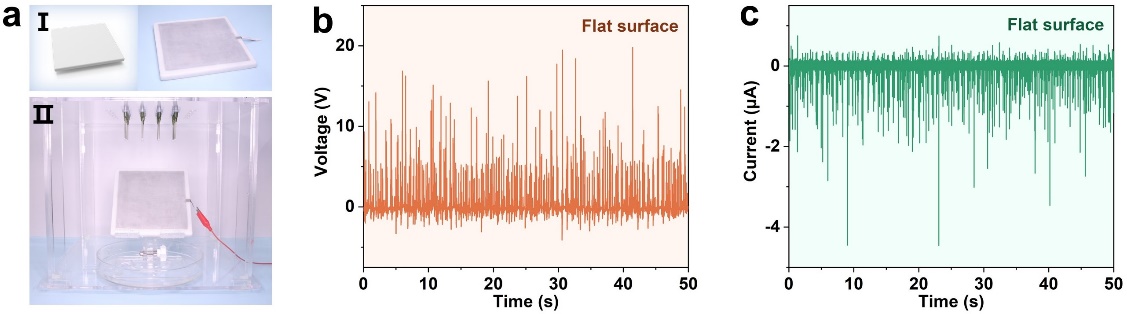


**Figure S17.** a) SS-TENG sample (10 cm × 10 cm) can fit on the flat surface (I) and collect droplet energy (II), and b) *V_OC_* and c) *I_SC_* of SS-TENG under the multiple droplets.


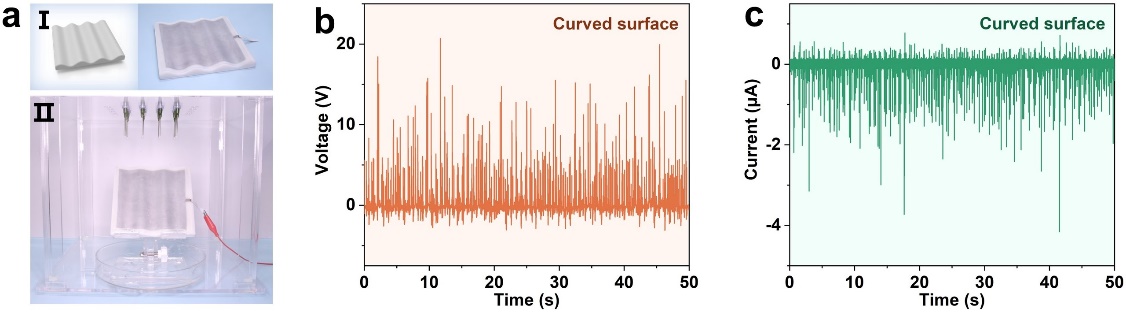


**Figure S18.** a) SS-TENG sample (10 cm × 10 cm) can fit on the curved surface (I) and collect droplet energy (II), and b) *V_OC_* and c) *I_SC_* of SS-TENG under the multiple droplets.


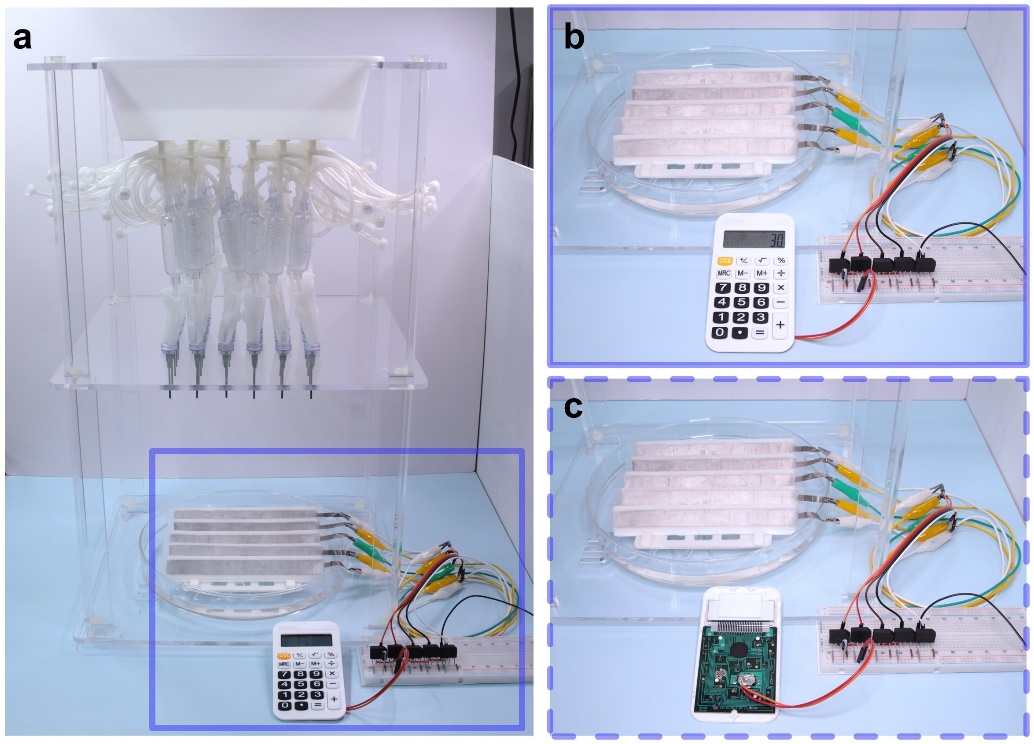


**Figure S19.** a) SS-TENG samples (150 mm × 15 mm × 5) are placed in a simulated rainy environment to collect droplet energy and power electronics. b) Enlarged views of the experimental circuit, and c) the back of the calculator.


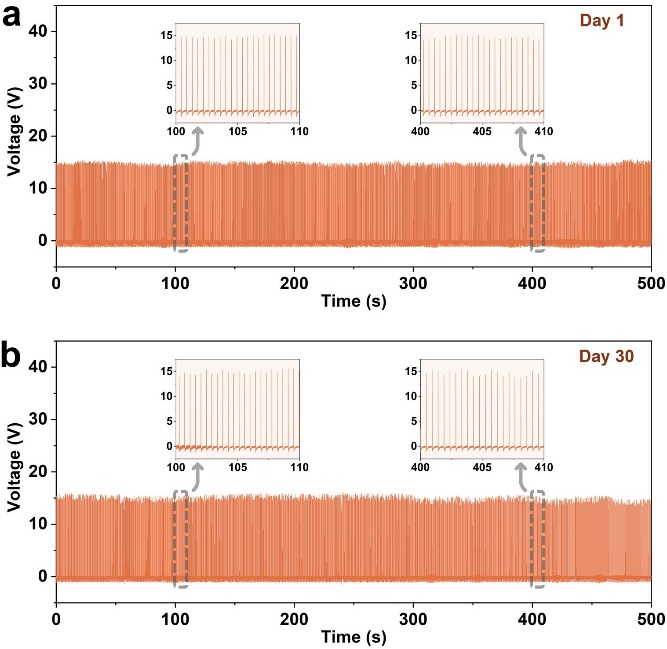


**Figure S20.** Long-time stability test of SS-TENG. *V_OC_* at a) day 1 and b) day 30. Inset: *V_OC_* at 100 s and 400 s, reflecting no decline in *V_OC_*.


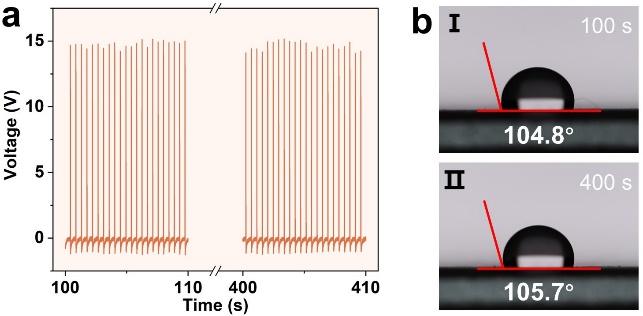


**Figure S21.** a) Comparison of *V_OC_* between the time intervals of 100 ~ 110 s and 400 ~ 410 s. b) Water contact angles of triboelectric layer after 100 s and 400 s of operation.

A comparison of the *V_OC_* between the time intervals of 100 ~ 110 s and 400 ~ 410 s in Figure 6h was conducted (Figure S21a). The *V_OC_* values remained within ranges of 14.2 ~ 15.1 V and 14.1 ~ 15.2 V for these two intervals respectively, showing no significant fluctuations. Additionally, the water contact angles of the SS-TENG’s triboelectric layer surface were measured after 100 s and 400 s of operation, yielding values of 104.8° (Figure S21a-I) and 105.7° (Figure S21a-II) respectively, with no observable variations.

**Movie S1.** Stretch-release possess of the triboelectric layer.

**Movie S2.** Statuses of the droplet on different triboelectric layers.

**Movie S3.** SS-TENG samples are placed in a simulated rainy environment to collect droplet energy and power a calculator.
